# The Monoterpenoid Perillyl Alcohol: Anticancer Agent and Medium to Overcome Biological Barriers

**DOI:** 10.3390/pharmaceutics13122167

**Published:** 2021-12-16

**Authors:** Thomas C. Chen, Clovis O. da Fonseca, Daniel Levin, Axel H. Schönthal

**Affiliations:** 1 Department of Neurological Surgery, Keck School of Medicine, University of Southern California, Los Angeles, CA 90089, USA; 2 Department of Neurological Surgery, Federal Hospital of Ipanema, Rio de Janeiro 22411-020, Brazil; clovis.orlando@uol.com.br; 3 Norac Pharma, Azusa, CA 91702, USA; dlevin@noracpharma.com; 4 Department of Molecular Microbiology & Immunology, Keck School of Medicine, University of Southern California, Los Angeles, CA 90089, USA

**Keywords:** blood–brain barrier, drug formulation, drug hybrids, intra-arterial delivery, intracranial malignancies, intranasal delivery, monoterpene, monoterpenoid, NEO100

## Abstract

Perillyl alcohol (POH) is a naturally occurring monoterpenoid related to limonene that is present in the essential oils of various plants. It has diverse applications and can be found in household items, including foods, cosmetics, and cleaning supplies. Over the past three decades, it has also been investigated for its potential anticancer activity. Clinical trials with an oral POH formulation administered to cancer patients failed to realize therapeutic expectations, although an intra-nasal POH formulation yielded encouraging results in malignant glioma patients. Based on its amphipathic nature, POH revealed the ability to overcome biological barriers, primarily the blood–brain barrier (BBB), but also the cytoplasmic membrane and the skin, which appear to be characteristics that critically contribute to POH’s value for drug development and delivery. In this review, we present the physicochemical properties of POH that underlie its ability to overcome the obstacles placed by different types of biological barriers and consequently shape its multifaceted promise for cancer therapy and applications in drug development. We summarized and appraised the great variety of preclinical and clinical studies that investigated the use of POH for intranasal delivery and nose-to-brain drug transport, its intra-arterial delivery for BBB opening, and its permeation-enhancing function in hybrid molecules, where POH is combined with or conjugated to other therapeutic pharmacologic agents, yielding new chemical entities with novel mechanisms of action and applications.

## 1. Introduction

The blood–brain barrier (BBB) is a highly selective semipermeable interface between the bloodstream and the brain parenchyma that acts as a gatekeeper to control the entry of substances from the systemic circulation into the brain. It not only manages the brain entry of endogenous substances present in the circulation, such as neuroactive solutes, nutrients, hormones, and antibodies, but also provides protection by restricting brain access by potentially harmful agents, such as metals and toxins. The influx and efflux of molecules are actively regulated with the prime objective to maintain brain homeostasis and protect this organ from harm [1,2]. For a detailed description of these mechanisms and their underlying anatomical elements, the interested reader is referred to excellent recent reviews [3,4].

By allowing necessary nutrients and other beneficial materials to enter the brain whilst allowing waste products to exit and preventing harmful substances from entering the brain, the BBB provides great benefit in protecting and ensuring optimal brain function. At the same time, however, this armor-like role of the BBB represents a double-edged sword because it can also block brain access by a large number of pharmacological agents that may be needed within the brain in order to effect their therapeutic action against brain-localized diseases. In fact, within the context of brain-metastatic spread from peripheral tumors of the lung, breast, melanoma, and some others, effective exclusion of therapeutic agents from the brain has resulted in the well-recognized paradox of an increasing incidence of brain metastases despite improvements in general clinical care and substantial progress in developing novel therapeutic agents [5,6,7]. For example, in the case of breast cancer subtypes that overexpress epidermal growth factor receptor 2 (EGFR2/HER2/Neu; generally called HER2+), the introduction of the humanized monoclonal antibody trastuzumab (Herceptin^®^) and other HER2-targeted agents resulted in significantly improved treatment outcomes for the peripheral disease. However, HER2+ breast cancer has a propensity to metastasize to the brain and these brain seeds cannot effectively be reached by therapeutic antibodies. As a result, while extracranial malignancy generally responds well to intravenous trastuzumab (and other HER2-targeted therapeutics), cancerous seeds in the brain are, for the most part, isolated from this systemic therapeutic intervention. Whilst treatment success in the periphery enables patients to live longer, cancer cells residing in the sanctuary of the brain consequently have more time to develop sizable metastases and eventually trigger cancer recurrence and a grim prognosis [5,6,7,8].

Secondary brain cancer as a result of metastatic spread from peripheral cancer types represents the most frequent intracranial malignancy. Primary brain cancers, malignant gliomas in particular, present with no better prognosis and their effective management also remains an inadequately met clinical need. For instance, glioblastoma (GBM, formerly known as glioblastoma multiforme; IDH wildtype, grade IV) is the most aggressive cancer that originates in the brain, with a median overall survival of 8–10 months from the time of diagnosis [9,10,11]. Patients with higher performance scores (based on functional impairment [12]) and overall greater physical robustness at the time of diagnosis are better predisposed to withstand the rigors of extensive and taxing therapies and, therefore, are more likely to experience an extended average survival of another 6–12 months. However, even with patients demonstrating higher Karnofsky performance scores (KPS), recurrence of GBM is nearly universal and the vast majority of patients eventually die from this insidious disease within just a few years at best.

The current standard of care for GBM consists of surgery (if possible), followed by chemoradiation, which consists of oral temozolomide (TMZ; Temodar^®^) combined with ionizing radiation. TMZ is a prodrug that releases diazomethane, which is an alkylating agent. The clinical use of TMZ for GBM was cemented by a landmark clinical study by Stupp et al. [13], showing that the combination of radiation therapy (RT) with TMZ for 6 weeks, followed by 6 months of TMZ alone, resulted in greater patient survival duration than was achieved by the use of radiation alone. It was, however, noted that the median survival benefit was small: inclusion of TMZ with the RT regime extended the average survival from 12.1 months (RT alone) to 14.6 months, i.e., a life expectancy increase of only 2.5 months. The fraction of patients surviving 5 years also showed an increase, from 1.9% for RT alone to 10.9% for the combination treatment [13,14]. While these improvements were validated by subsequent studies, it remains noteworthy that clinical trials generally harbor selection bias, usually favoring patients with higher performance scores and, therefore, patients in ordinary clinical practice may not always be able to receive or tolerate the full spectrum of therapeutic interventions. As such, the publicized survival data derived from most clinical trials represent a somewhat skewed picture as compared to the entire cohort of GBM patients, and improved therapies are still urgently needed [15].

Similar to the above example of trastuzumab, where the BBB severely limits access of this therapeutic antibody to its intracranial HER2+ targets, the BBB significantly interferes with TMZ brain entry as well. Although TMZ is generally considered BBB permeable, neuropharmacokinetic measurements in patients with primary or metastatic brain tumors established a brain-to-plasma TMZ concentration ratio of only 0.2 [16,17], indicating that in practice, only a small fraction of systemically available TMZ enters the brain. This low amount entering the brain is not entirely surprising because TMZ was not specifically developed as a brain-targeting agent.

Within malignant brain lesions, the organization and function of the BBB can be altered due to pathological changes caused by tumor cells, including extensive neo-angiogenesis, which is particularly prominent in GBM tissue. Such structural alterations are generally referred to as the blood–tumor barrier (BTB), which is located between brain tumor tissue and adjacent capillary vessels [18,19]. It was observed that the barrier function of a BTB is compromised and not as effective as a normal BBB, a phenomenon called leakiness. This may explain why some chemotherapeutic drugs, such as etoposide, cisplatin, methotrexate, or cyclophosphamide, which are known to not effectively penetrate the BBB, can achieve occasional therapeutic responses in the brain when given at higher dosages [20,21]. The severity of barrier breakdown is, however, highly heterogeneous, both in primary and secondary brain malignancies. For example, a detailed preclinical analysis [22] of 2000 individual lesions of breast–brain metastases in mice demonstrated large differences between individual lesions—and even within the same lesion—with regard to BBB/BTB permeability. Although 90% of these lesions showed barrier compromise, their uptake of different clinically used chemotherapeutic drugs was less than 15% of that of other tissues or peripheral metastases. Clinical studies in GBM patients that used contrast-enhanced magnetic resonance imaging (MRI), positron emission tomography (PET) imaging, and analysis of surgically removed tissues also established significant heterogeneity: while some tumor regions present with deteriorated BTB, others display an intact barrier [23,24]. Therefore, despite being partially compromised within brain tumor lesions, the BTB is still operational enough to substantially impede effective drug delivery and prevent therapeutic activity. Despite the presence of more or less “leaky” sections of the BTB, effective drug delivery requires consideration of the intact BBB for reliable and more broadly applicable therapeutic benefit. 

Several diverse strategies are being explored to increase drug penetration of the BBB and achieve efficient brain entry of therapeutic agents. Some of these are highly invasive procedures that may require surgery and hospitalization of patients, such as intrathecal and intraventricular injection, convection-enhanced delivery by direct injection, or implantation of gels, wafers, and microchips for localized release, all of which seek to circumvent the BBB and place therapeutic agents directly into the brain or tumor tissue. Transient opening of the BBB by intracarotid infusion of mannitol or chemical disruptors (e.g., leukotrienes, bradykinins) represents another invasive method that can facilitate brain access for drugs present in the systemic circulation. All of these procedures are, however, associated with significant risks, such as vasogenic brain edema, embolism, seizures, and neurological deficits, as well as high costs [19,25,26].

There is a great need then for less invasive procedures that allow for effective drug access to brain-localized malignancies. Among the strategies that are being pursued to achieve this goal are novel interventions that reversibly open the BBB, drug modifications that enable more efficient penetration of the BBB, or intranasal delivery with the goal to achieve direct nose-to-brain drug transport to circumvent the BBB altogether. In the discussion that follows, we review progress in this field that was achieved with the use of perillyl alcohol (POH), a naturally occurring monoterpenoid related to limonene [27], and with NEO100, an engineered version of this monoterpenoid that features ultra-high purity and is being produced under Current Good Manufacturing Practice (CGMP) regulations. NEO100 has revealed multifaceted applicability and uses that include intranasal delivery (either alone in monotherapy fashion or as a facilitator of enhanced permeability for other drugs), intra-arterial delivery to reversibly open the BBB, and as a covalently attached modifier for other drugs to achieve superior penetration, not only of the BBB but also of other critical barriers, the cell membranes (cytoplasmic and nuclear), and the skin. Below, we provide a detailed description of the physicochemical properties of POH and NEO100, followed by a review of their versatile applications to overcome biological barriers and allow for improved therapeutic access to malignant lesions.

## 2. The Monoterpenoid Perillyl Alcohol (POH)

### 2.1. Biochemical Description of POH

**Scheme 1:**
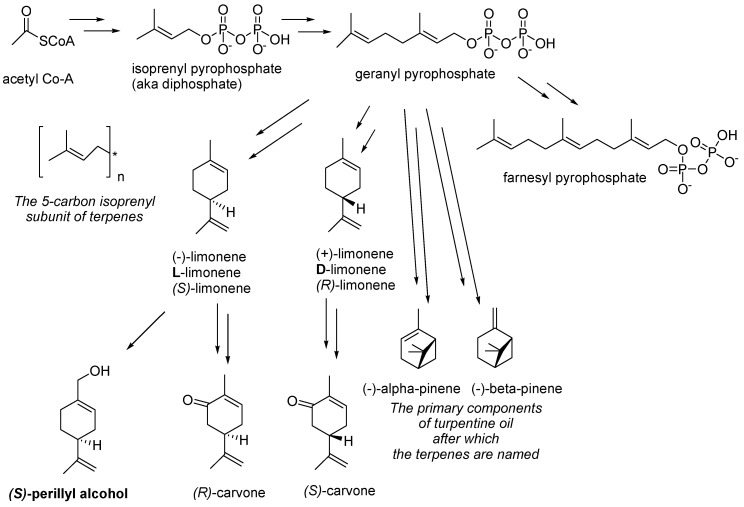
Biosynthesis of some terpenes and terpenoids. The biosynthesis of terpenes proceeds via the isoprenyl building block to assemble higher-order geranyl (2× isoprenyl moieties), farnesyl (3× isoprenyl moieties), and other intermediates en route to terpenes, such as limonenes (in citrus oils) and pinenes (in pine oils), and further functionalized (e.g., oxidized) terpenoids, such as carvones and (S)-perillyl alcohol.

POH was first extracted from herbs of the genus *Perilla* [28], from which it derived its name, and to various extents is a constituent of essential oils from a variety of botanicals, including peppermint, spearmint, lavender, bergamot, lemongrass, sage, caraway, thyme, rosemary, celery seeds, cherries, and cranberries [27,29]. Common synonyms of POH are perilla alcohol, perillol, and p-mentha-1,8-dien-7-ol, among others. Its IUPAC name is (4-prop-1-en-2-ylcyclohexen-1-yl)methanol. It is a terpene (or, more accurately, a terpenoid, as it is a terpene modified by derivatization through the addition of a hydroxyl group). The terpenes were so-named in 1866 by the German chemist August Kekulé from the name of the pine-oil extract turpentine [30], which is primarily comprised of terpenes, a classification expanded to include tens of thousands of chemical substances made up of repeating 5-carbon isoprenyl subunits (see Scheme 1 for an overview of some terpene biosynthetic pathways) [31].

POH itself is a fragrant oil with a somewhat heavy, floral, lavender-like fragrance that is used in perfumes, cosmetics, and some cleaning products. Other reported uses are as an ingredient in baked goods, frozen dairy, gelatine, puddings, beverages, and candies [32]. It is, however, now also of increasing interest as a therapeutic agent in the prevention and treatment of cancers (details below). POH is a chiral compound (i.e., one whose 3-D shape is not superimposable on its mirror image “enantiomer”). The naturally occurring form of POH is primarily the enantiomer with (S)-stereochemistry at the chiral center (which is the carbon atom having the substituents that are non-superimposable on their mirror image) (Scheme 1). Although the biological activity of the other mirror image (R)-enantiomer is, as yet, untested, most biological components and most biochemical processes involve single enantiomer stereochemistry such that it is likely that different enantiomers will interact differently with biological receptors and pathways. This is evident, for example (Scheme 1), with the terpenoid (R)-carvone (from spearmint oil), which, unsurprisingly, smells of spearmint, whilst its enantiomer (S)-carvone (from caraway or dill seed oil) exhibits the totally different odor of rye bread (which is flavored by caraway seeds) due to the carvones’ stereochemically differentiated interactions with the chiral olfactory receptors in our nasal cavities [33,34].

Scheme 1 shows the biosynthesis and chemical structures of these aforementioned terpenoid materials. Note that the stereochemistry in this reaction scheme (for example illustrated by the limonene enantiomer intermediates in the biosynthesis of POH and carvones) may be designated by (+)/(−), D/L, or (R)/(S), which can be confusing. (R) and (S) are determined from the 3-D chemical structure, where (R) denotes the enantiomer with the hydrogen (H) substituent on the chiral carbon atom oriented to lie backward away from the viewer and the other substituents arranged clockwise in descending order of priority (atomic weight), whilst the (S) enantiomer is the mirror image of the (R) enantiomer. The D designation refers to the stereochemistry derived by chemical synthesis from D-glyceraldehyde and vice versa for L. Meanwhile, the (+) or (−) refer to the direction of rotation of plane-polarized light by a solution of the material where (+) implies clockwise rotation of plane-polarized light and (−) denotes the opposite. Suffice to say that there is no reliable general rule to predict the correlation of (R) vs. (S) with D or L and with (+) or (−) [35].

POH is classified as a cyclic monoterpenoid (where the monoterpene designation refers to its biosynthesis from a single geranyl pyrophosphate moiety, shown in Scheme 1, followed by oxidation of the terpene backbone while catalyzed by cytochrome P450 enzyme to achieve the terminal hydroxylation; hence, the designation “terpenoid” rather than “terpene”). Its molecular weight is 152.2 g/mol, which is about 2.5 times the molecular weight of, for example, acetic acid in vinegar; therefore, it is a lot less volatile than acetic acid (boiling point 118–119 °C at atmospheric pressure), with a consequently higher boiling temperature of 244 °C at atmospheric pressure, but it still exerts sufficient vapor pressure and active interaction with our olfactory biochemistry to be easily detected by smell. The polar hydroxyl functionality makes the molecule more hydrophilic (i.e., water miscible) than non-hydroxylated equivalent terpenes, such as pinenes, which are water immiscible, such that POH does demonstrate some limited solubility in water (around 1.9 mg/mL) and a mix of lipophilic (fat solubility) and hydrophilic (water solubility) properties. It is this biphilic (or amphiphilic) nature (both fat and water affinity) that perhaps explains the remarkable properties of POH in achieving essentially free passage throughout the body, across the blood–brain barrier, and across cell membranes to realize its therapeutic potential and to potentiate the therapeutic potential of other materials with which it is partnered.

**Scheme 2:**
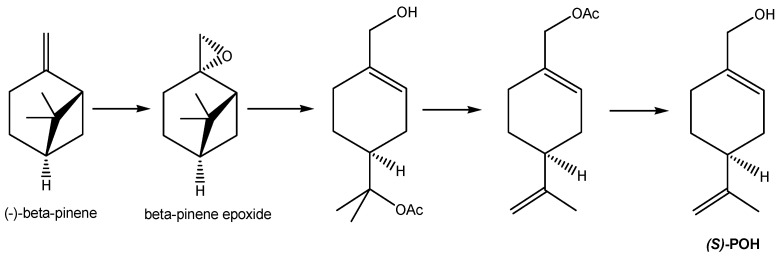
Semi-synthetic manufacture of POH. An example of semi-synthetic industrial manufacture of POH from pine oil, whereby (-)-beta pinene is epoxidized by treatment with peracetic acid; the beta-pinene epoxide is then rearranged with sodium acetate in acetic acid to give POH with acetic acid added across the isopropylene substituent, and acetic acid is subsequently eliminated via pyrolysis. A final hydrolysis step gives (S)-POH.

**Scheme 3:**
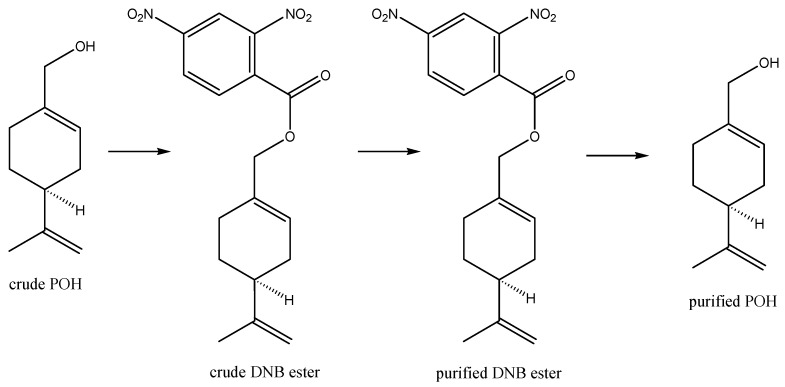
Purification of POH for pharmaceutical use. Commercial or industrial POH can be further purified from contaminating related substances for pharmaceutical use by converting the POH to a solid dinitrobenzoate ester derivative, which can be purified via recrystallization and isolation from the impurities left in recrystallization mother liquors before the purified nitrobenzoate ester is then hydrolyzed to give purified POH, from which the unwanted dinitrobenzoic acid is removed via aqueous extraction under alkaline conditions. This extra purification step leads to the synthesis of NEO100 (NeOnc Technologies, Los Angeles, CA, USA).

Whilst nature does an astonishingly efficient job at converting carbon dioxide, water, sunlight, and air to POH via biosynthesis in plants, the POH so generated is present in a cocktail with other products; as such, this POH is unsuitable for pharmaceutical use without costly and extensive purification. This is because regulatory approval for pharmaceutical use requires consistent high-purity composition to verify safety and efficacy, without risk of any changes in composition that could introduce toxicity risks or variation in therapeutic efficacy that may imply potential risks to patients. Industrial manufacturing of POH therefore generally relies upon a combination of biosynthetically produced high-purity, low-cost (-)-beta-pinene, with subsequent industrial manufacturing conversion of this to POH, i.e., utilizing a “semi-synthetic” preparation. An example is outlined in Scheme 2 [36]. Chemistry along these lines is used for the industrial manufacture of POH at around 95% purity for perfume and antimicrobial purposes. Furthermore, the ability of bacterial enzymes to modify the monoterpene backbone has been exploited for the production of “natural” aromas for the flavor and fragrance industries, where suitably genetically engineered bacterial strains are used as a source of POH and other known and novel monoterpene derivatives [37,38,39]. For pharmaceutical use, however, even higher purity and consistency of composition are ideally required so that derivatization of POH is developed to generate a crystalline dinitrobenzoate ester intermediate (which is more easily purifiable by crystallization), followed by hydrolysis to release the purified POH (Scheme 3) [40]. The POH manufactured in this way was named NEO100 and is undergoing clinical trials for the treatment of glioblastoma (see details in sections below). 

### 2.2. Recognizing POH’s Potential for Anticancer Purposes

The earliest studies that explored the potential of POH for anticancer purposes were spearheaded by the team of Michael Gould, Jill Haag, Pamela Crowell, and others at the University of Wisconsin–Madison in Madison, Wisconsin [41,42,43]. Using a rat carcinogenesis model with 7,12-dimethylbenz(a)anthrazene (DMBA)-initiated mammary tumors, it was shown that 2% POH mixed with a daily diet (*w*/*w*) over 10 weeks resulted in regression of the majority of small and advanced mammary carcinomas [43]. Of note, the authors compared the activity of POH side by side to that of limonene and observed greater activity of the former, which they ascribed, at least in part, to differences in metabolic pharmacokinetics, where an acute dose of 2% POH achieved 2–3 times higher levels of terpene metabolites (primarily perillic acid and dihydroperillic acid) in the plasma of the treated rats as compared to rats fed 10% limonene. Similarly, when POH and limonene were dosed chronically at the same levels, the terpene metabolites were measured at >10-fold higher plasma levels in the case of POH. In parallel studies that compared the inhibitory activity of POH and limonene on the isoprenylation of small G proteins (details below), it was found that POH was greater than fivefold more potent than limonene [42]. Therefore, despite numerous earlier reports on the oncotherapeutic properties of limonene [44,45,46,47], these newer studies favored POH for further development and clinical trials (details below). 

### 2.3. Mechanisms of POH Anticancer Function

The first cellular target of POH to be identified was the process of isoprenylation of small G proteins, where POH and, to a lesser extent, limonene were found to inhibit small G protein isoprenylation, along with restricted cell proliferation [41,42,48,49]. In particular, the inhibition of farnesylation of p21-Ras generated excitement in view of the recognized critical role of this proto-oncoprotein in cell growth regulation and carcinogenesis [50,51,52]. A critical step during the activation process of Ras is its posttranslational modification via farnesylation, providing an anchor for attachment of the protein to the inner leaflet of the cytoplasmic membrane, which is critically required for the protein’s mitogenic and oncogenic activity [53]. The discovery that this process could be inhibited by POH provided early insight into how this monoterpenoid might interfere with tumor cell growth [48,51,54]. Besides Ras, however, numerous additional targets were recognized over the years that followed and it appears that the anticancer function of POH might be derived from its influence over a variety of different targets and processes in cancer cells. 

Several studies reported on the ability of POH to restrict the cell cycle progression of tumor cells through its effects on components of the cell cycle machinery. For instance, treatment of cells with POH in vitro results in the downregulation of various cyclin proteins, which represent the regulatory subunits of cyclin-dependent kinases (CDKs) whose activity is essential for cell cycle progression. Conversely, POH stimulates the expression of CDK-inhibitory proteins p15 (INK4b, *CDKN2B*), p21 (WAF/Cip1, *CDKN1A*), and p27 (Kip1, *CDKN1B*), all of which are able to block CDK activity [55,56,57,58]. The ensuing cell cycle arrest prevents tumor cell proliferation in vitro, translating to the inhibition of tumor growth in vivo. 

A host of other cellular targets of POH were identified, including immediate-early genes c-Fos and c-Jun (which heterodimerize to form transcription factor AP1) [55,59]; telomerase reverse transcriptase (hTERT) [60,61]; eukaryotic translation initiation factors eIF4E and eIF4G, along with their binding partner 4E-BP1 (key components of the translational cap-binding complex) [62,63]; sodium/potassium adenosine triphosphatase (Na/K-ATPase) [64,65]; Notch (which plays a role in tumor cell invasion and metastasis) [66]; nuclear factor kappa B (NF-κB) [67,68,69]; mammalian target of rapamycin complex (mTORC) [61,70]; and transforming growth factor beta (TGFβ) [55]. Furthermore, POH was characterized as an effective trigger of endoplasmic reticulum (ER) stress, which was revealed through its ability to stimulate the expression of two ER stress markers, CCAAT/enhancer-binding protein homologous protein (CHOP, also known as GADD153) and glucose-regulated protein of molecular weight 78 kDa (GRP78, also known as BiP) [71]. CHOP represents the key pro-apoptotic effector of ER stress [72], and its prominently increased expression in response to the treatment of tumor cells with POH is consistent with resulting cell death. The tumor-selective impact of POH (and of other agents that are able to aggravate cellular stress levels) via the ER stress response was conjectured to be based on pre-existing, chronic stress levels in tumor cells, which are lacking in normal cells. In tumor cells, aggravated stress reaches the maximum tolerated threshold quicker, whereas normal cells have more leeway to accommodate and therefore tolerate these increased levels [73,74].

While a plethora of cellular POH targets was identified, the decisive contribution of each one has not yet been clearly established. It is likely that some targets are more critical than others, and cell-type-specific availability of some targets might factor in as well. Furthermore, in view of multiple alterations that are generally acquired during the carcinogenic process of tumor cell development, a pleiotropic impact on tumor cell functions might in fact be desirable and beneficial to achieve improved therapeutic outcomes.

## 3. Clinical Evaluation of Oral POH

Based on the well-documented, highly promising anticancer functions of POH that were characterized in a variety of preclinical in vitro and in vivo models, the agent was moved forward to clinical trials in humans. In the first of these trials [75], 18 patients with advanced malignancies received POH that was formulated in soft gelatin capsules containing 250 mg POH mixed with 250 mg soybean oil. Dosing was by mouth three times a day, every day, at escalating doses of up 2400 mg/m^2^/dose (approximately 4 g POH in 16 capsules per dose for a 60 kg patient; 12 g total per day). POH was generally tolerated, with gastrointestinal (GI) toxicities as the most frequent events, presenting as nausea, belching, vomiting, unpleasant taste, and early satiety, which were correlated with increasing dose levels and proved dose-limiting. Pharmacokinetic plasma measurements were able to quantify the major metabolites perillic acid and dihydro-perillic acid [75] but did not detect the parent compound POH, consistent with earlier studies in POH-fed rats that easily detected the terpene metabolites but not POH itself [43]. The metabolite half-lives were approximately 2 h and there was no evidence of drug or metabolite accumulation [75], which spawned the rationale to spread drug administration more evenly in the hopes of achieving greater exposure by increasing the dosing frequency. Therefore, in a subsequent Phase I trial, the dosing frequency was increased to four times a day at up to 1600 mg/m^2^/dose [76]. Similar GI toxicities as before were noted, and the maximum tolerated dose of POH was determined as 1200 mg/m^2^/dose (approximately 8 g total per patient per day from 32 capsules per day), which was recommended as the starting dose for Phase II studies. 

Several Phase II trials with oral POH followed in cohorts of patients with advanced ovarian cancer [77], metastatic prostate cancer [78], metastatic breast cancer [79], metastatic androgen-independent pancreatic cancer [80], and metastatic colorectal cancer [81]. Dosing was four times each day for an initially planned 6-month period. As in the previous Phase I studies, the toxicity was mild to moderate and primarily limited to GI effects and fatigue, although there was significant variability in patient responses. Overall, however, the therapeutic activity of POH was unimpressive and mostly disappointing. Furthermore, while GI toxicity was not unacceptably severe based on common clinical criteria, the chronic and unrelenting nature of these effects caused problems with patient tolerance and compliance to the point where some patients discontinued their participation in these trials [77,78,81]. These results dampened the enthusiasm that was generated by the highly promising preclinical studies of POH and, so far, no Phase III clinical trials of orally administered POH have been initiated [82]. A newer route of administration, dosing POH intranasally rather than orally (so as to avoid the GI-related side effects), has revived some of the lost excitement and has sparked new hope that POH might still find its place among new and improved clinically used cancer therapeutics. The efforts to establish intranasal POH as a beneficial cancer treatment, in particular for cancer types in the brain that are protected by the BBB, are described below.

## 4. Intranasal Perillyl Alcohol

Intranasal drug delivery represents a non-invasive route of administration with several advantages over oral drug delivery. Although the extent of these advantages may differ significantly, based on the specifics of the delivery methods, drug formulation, and physicochemical properties of the active pharmacological ingredient (API), they usually include quicker biological availability, avoidance of hepatic first-pass metabolism, and potentially less systemic side effects. Moreover, the potential for direct nose-to-brain transport offers the prospect of increased central nervous system (CNS) drug availability by side-stepping the BBB [83,84,85]. 

**Figure 1:**
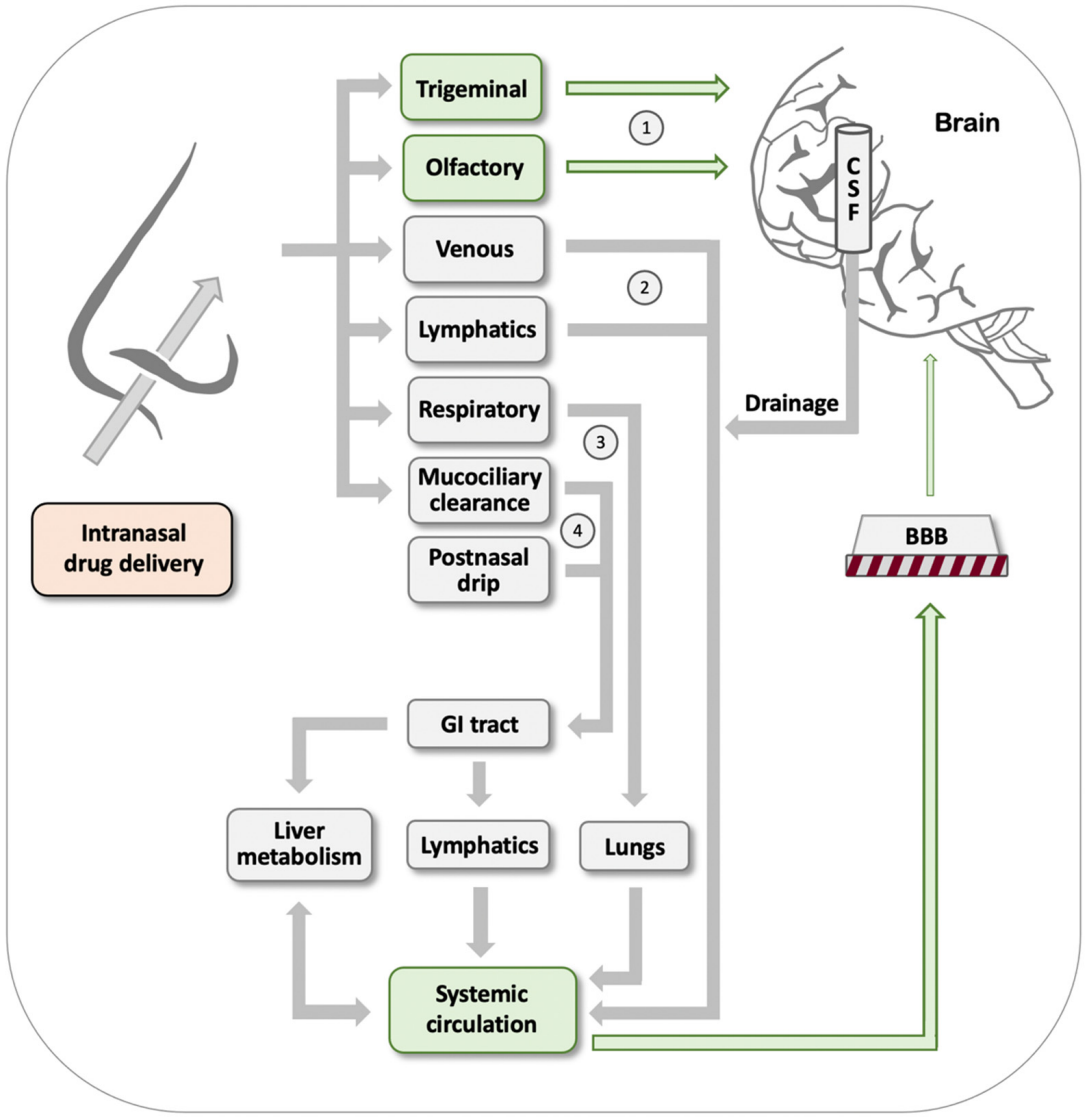
Available routes for intranasal drug delivery. After intranasal delivery, drugs can potentially reach the brain through several quite distinct routes: either through direct nose-to-brain transport via the trigeminal and olfactory nerves (labeled as 1), or indirectly via first entering the bloodstream through the mechanisms labeled as 2–4. In the latter routes, however, drugs will encounter the BBB, which prevents efficient brain entry of the vast majority of pharmaceutical agents. See the text for further details.

As outlined in Figure 1, after drugs have entered the nasal cavity, there are four main routes by which such drugs may reach the brain [83,86,87,88]: (#1) Direct nose-to-brain transport is offered through drug absorption by the olfactory bulb and through drug uptake by branches of the trigeminal nerve. This pathway is of particular interest for purposes of delivering drugs aimed at the brain, because it does not involve the drawbacks of having to overcome the BBB. (#2) Extensive vascularization of the nasal epithelium and a rich lymphatic network are conducive to effective drug uptake that will deliver the drug into the systemic circulation. However, subsequent brain uptake by this path will be subjected to the limitations of having to cross the BBB. (#3) Intranasally delivered drugs may also be inhaled or aspirated into the lungs, in particular when APIs are formulated as aerosols. From here, they effectively enter the bloodstream, only to again encounter the BBB as an obstacle on their way to the brain. (#4) Postnasal drip and mucociliary clearance result in drug transport to the GI tract. Depending on the lipophilicity of the drug and other properties, the API may continue through the portal vein and liver, or via intestinal lymphatic transport. The route via the portal vein poses the additional challenge of substantial drug metabolism, which may result in effective drug inactivation. In both these cases, the drug ends up in the systemic circulation and encounters the BBB.

Comparing the above four pathways illustrates the potential advantages of intranasal drug delivery. Only the first one, i.e., direct nose-to-brain transport, has the potential for direct delivery to the brain without encountering the obstacle placed by the BBB. While the efficiency of this route is very low in general and can be as low as 0.01–1% of the oral dosage [83] (depending in large part on biopharmaceutical formulation and physicochemical properties of the API [88,89]), this small amount can nevertheless prove advantageous over oral delivery due to avoidance of potentially inefficient absorption and elimination in the GI tract, hepatic first-pass metabolism, and dilution and protein binding in the bloodstream. Furthermore, several oral CNS drugs barely penetrate the BBB and, therefore, must be given at increased dosages that raise the risk for adverse effects in the periphery. This latter consideration applies to parenteral injections of drugs as well, further emphasizing the potential advantages of intranasal delivery. For further reading, see the following excellent reviews: [83,84,90,91].

### 4.1. Intranasal POH as Monotherapy

While several Phase I and II clinical trials of oral POH, as described above, yielded disappointing results and never moved into Phase III [92], a case report published in 2006 [93] stimulated renewed interest in POH by way of intranasal delivery. This report described a 62-year-old woman in Brazil with histologically confirmed anaplastic oligodendroglioma, a malignant grade III glioma. Despite standard combination therapy with surgery, radiotherapy, and chemotherapy, she presented with a second recurrency, at which time, intranasal POH (obtained from Sigma-Aldrich/MilliporeSigma, St. Louis, Missouri, USA) was initiated at 0.3% concentration and four times daily dosing. After 5 months of treatment, a follow-up MRI revealed regression of the tumor [93].

**Figure 2:**
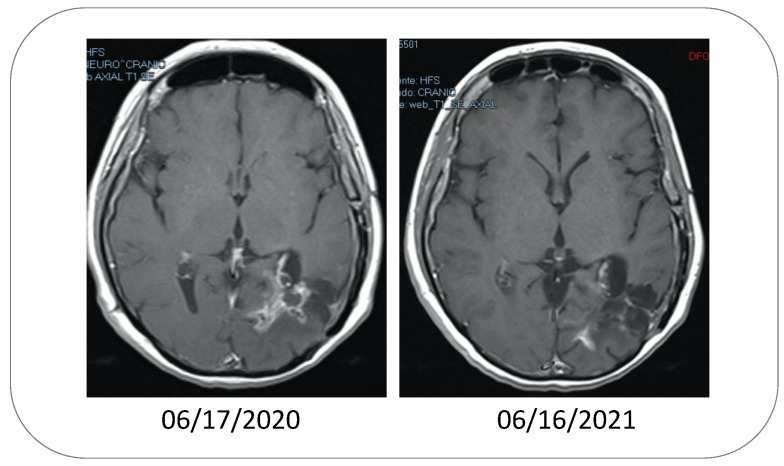
MRIs showing the effect of inhaling POH in a patient with relapsed GBM. Note the tumor tissue in the initial MRI on 06/17/2020, as indicated by intense non-homogeneous enhancement. One year later (06/16/2021), the lesions presented with reduced dimensions and a marked reduction in contrast enhancement, suggesting a response to the POH treatment. While these MRIs present an illustrative example derived from the group of patients who responded favorably to intranasal POH and achieved stable disease status, not all patients responded similarly well, with some patients progressing after the initial response, despite continuous treatment.

Based on this positive outcome, intranasal POH delivery was further investigated in additional relapsed glioma patients. A follow-up study reported on a small cohort of 37 patients with recurrent high-grade glioma, where the same four-times-daily POH regimen was administered intranasally (55 mg per dose for 220 mg total per day) [94]. Here as well, it was reported that this novel treatment strategy resulted in encouraging outcomes, with decreased tumor size (Figure 2) and increased survival in several patients. Further expansion of these studies included several hundred recurrent patients with malignant glioma, including GBM, and dosing was further increased to 133 mg per dose (533 mg/day) [95,96]. Overall, these Phase I/II studies further indicated the encouraging therapeutic activity of intranasal POH, with numerous patients remaining under clinical remission even after several years of exclusive POH treatment. Of note, despite the four-times-daily dosing, this regimen was very well tolerated and adverse events were almost non-existent, contributing to very high patient compliance [96].

The above studies were performed in Brazil and are in part still ongoing. Despite the inclusion of hundreds of patients and numerous encouraging observations, limitations include the absence of an independent Contract Research Organization (CRO) for external clinical data management and trial monitoring. Inspired by the promising Brazilian experience, a Phase I/IIa study with recurrent GBM patients was initiated in the United States (ClinicalTrials.gov identifier: NCT02704858) and the results from the Phase I part were published in early 2021 [97]. For this trial, POH had to be produced for pharmaceutical use under CGMP regulations, which yielded a highly pure (>99%) product that was labeled NEO100 (see Scheme 3). The dose finding in Phase I used four cohorts of three patients each and escalated the four-times-daily dose up to a maximum of 288 mg, for a total of 1152 mg/day, which represented roughly double the dosage used in the Brazilian trials. 

This new study [97] found that intranasal NEO100 was very well tolerated, consistent with the earlier Brazilian trials’ findings. Among the 12 patients in Phase I, there were no grade 3 or 4 adverse events (based on NCI Common Terminology Criteria for Adverse Events [98]). Among the few lower-grade events defined as “definitely treatment-related” were rhinorrhea, skin irritation around the nose, nasal pruritus, and cough, which were observed in a minority of patients. Intriguingly, the frequency of these events did not correlate with increased dosages and, in fact, most of them were observed at the lowest dosage. It was concluded that intranasal delivery of NEO100 to recurrent GBM patients is safe [97] and free from the problematic side effects suffered from oral dosing.

**Figure 3:**
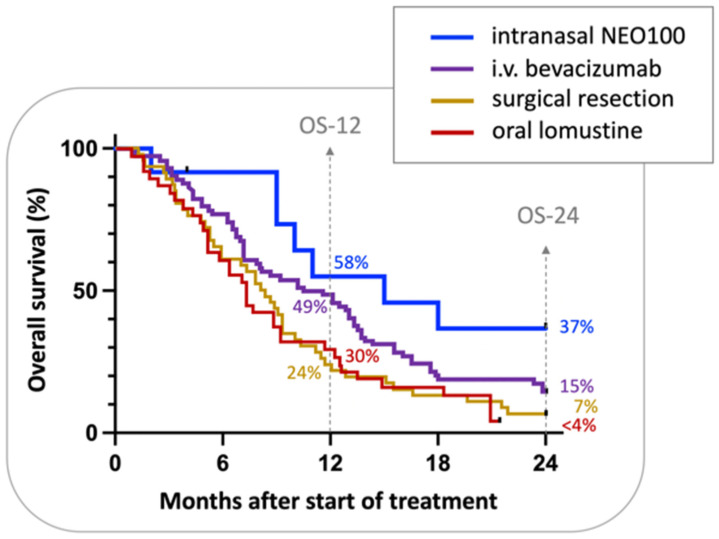
Comparison of the overall survival of recurrent GBM patients. Shown are the Kaplan–Meier curves representing the probability of survival of recurrent GBM patients undergoing different treatment regimens. The blue line indicates results for intranasal POH/NEO100 treatment (n = 12) obtained from a Phase I study [97]. The purple line represents a single-institution retrospective of the clinical real-world experience with bevacizumab (n = 74) [102]. The gold line is a historical comparison from 1998 that monitored the survival of patients after surgical resection of recurrent tumors (n = 46) [99]. The red line represents data from a randomized Phase III trial published in 2013 [100], where patients (n = 65) received lomustine. Percentages shown in the graph refer to the OS-12 and 2-year survival (OS-24) of the different treatment regimens after diagnosis of recurrence.

Although Phase IIa of this trial is still ongoing, further analysis of Phase I data sought to provide preliminary insight into the activity of intranasal NEO100, which was determined based on radiographic responses, i.e., magnetic resonance imaging (MRI) performed every two months. Despite the small cohort of only 12 patients, there were intriguing signs of therapeutic benefit. Overall survival at 12 months (OS-12) was 55%, median OS was 15 months, and two-year survival was 37%. These results compared favorably with historical controls and other recent trials with cohorts of recurrent GBM patients (Figure 3) [99,100,101,102]. For example, a 2018 retrospective [102] reviewed the clinical experience with bevacizumab, an anti-angiogenic monoclonal antibody that is approved for recurrent malignant glioma [103]. This analysis showed that therapy with bevacizumab yielded an OS-12 of 49%, median OS of 11 months, and two-year survival of 15%. A randomized Phase III trial with 65 recurrent GBM patients who received lomustine (also known as CCNU, a nitrosourea-based DNA alkylating agent commonly used in the recurrent GBM setting [104]), yielded an OS-12 of 30% and 2-year survival of below 4% [100]. Among such comparisons, it remains noteworthy to emphasize the lack of serious adverse events with the use of intranasal POH/NEO100, which is not the case for the majority of other commonly used therapies applied to recurrent GBM. Thus, even if there are only relatively small increments in survival in comparison to other regimens, quality of life (QoL) issues need to be factored into treatment decisions as well. Furthermore, maintenance of QoL for longer periods may increase the chances that further salvage therapies could be considered subsequent to a later recurrence (given enhanced patient strength and robustness for additional therapies compared with the outcomes after more debilitating conventional cytotoxic treatments).

As with most cancer therapies, efforts are underway to establish predictors of response to intranasal therapy with POH. One such study indicated that patients with recurrent GBM and treated with intranasal POH survived significantly longer when tumors were located deep in the basal ganglia as compared to lobar localization [105]. Another study provided preliminary evidence that those recurrent GBM patients harboring a hypermethylated tumor phenotype and preferential concurrent polymorphism for the thymidine-thymidine (TT) variant of *rs1801133* of the methylene-tetrahydrofolate reductase (MTHFR) gene appeared to respond more favorably to intranasal POH, as indicated by prolonged survival [106]. Furthermore, isocitrate dehydrogenase 1 (IDH1) and IDH2 are emerging as potentially useful markers [107]. Specific mutations in either of these genes result in a neomorphic phenotype of the encoded protein, where enzymatic activity switches from the production of alpha-ketoglutarate to the synthesis of D-2-hydroxyglutarate [108]. In the case of newly diagnosed malignant glioma, such mutations in either IDH1 or IDH2 result in a better prognosis irrespective of the selected treatment approach [109], although this has not yet been firmly established for recurrent GBM [110]. Data from the above-described Phase I trial of NEO100 were separated into IDH-wild type and IDH-mutant cases, which revealed pronounced survival advantage for those intranasal NEO100-treated patients that harbored IDH-mutant tumors [97]. Due to the small cohort in this study, it remains to be established whether this survival advantage is primarily based on IDH status (and possibly enhanced by NEO100) or whether intranasal NEO100 contributes the decisive benefit. The currently ongoing Phase IIa part of this study is expected to provide clarification. 

In the meantime, other ongoing studies are building on the highly promising results obtained with intranasal POH by exploring respective combination approaches. For instance, a case study [111] reported a 51-year-old woman with inoperable GBM who had recurred after standard treatment with TMZ-based chemoradiotherapy and subsequently was enrolled in a clinical trial of intranasal POH concomitant with oral TMZ. After initiation of POH + TMZ, MRI scans revealed a marked reduction of the initially enhanced lesion, and no recurrences were noted over the following two years. This provided an encouraging example where the combination approach appeared to demonstrate benefit in this case of non-resected recurrent GBM. Other efforts sought to explore the combination of intranasal POH with dietary interventions. In one such study [112], 32 recurrent GBM patients were enrolled and divided into two groups, one where intranasal POH was combined with a ketogenic diet (KD) and one where intranasal POH was given without any specific dietary intervention. After 90 days of treatment, the average tumor area in the combination group showed greater reduction as compared to baseline at time 0, suggesting more effective therapeutic activity. A similar approach, this time with 29 recurrent GBM patients and combined with a low carbohydrate diet (LCD) also showed a trend for the greater activity of intranasal POH when combined with an LCD [113]. 

Collectively, the above studies established POH as the best-studied intranasally delivered anticancer agent. As of 2021, there are patients who have been using this regimen uninterrupted for many years, indicating the prospect that this form of circumventing the BBB might be able to convert a deadly disease to a chronic one, at least in some of the patients. 

Among the challenges of studying brain-targeted delivery of POH has been the difficulty of performing neuro-pharmacokinetic measurements of this compound. As mentioned above in the context of clinical trials with oral POH, PK measurements of blood levels relied on the quantification of the stable metabolite perillic acid because POH was not detectable with the analytical methods used. However, more sensitive and optimized approaches have become available. For instance, Zhang et al. reported on their detection of intact POH and perillic acid in plasma from a patient after oral delivery of POH using gas chromatographic–mass spectrometry (GC-MS) [114]. In studies with mice or rats, other groups applied ultra-performance liquid chromatography/tandem mass spectrometry (UPLC-MS/MS) [115] or high-performance liquid chromatography (HPLC)–ultraviolet (UV)-based approaches [116] to quantitate POH (and perillic acid) in the plasma and lungs of animals after intranasal delivery of POH. In a very recent study that investigated the impact of intranasal POH in a mouse model of cerebral malaria, the authors succeeded in quantifying POH in brain tissue using GC-MS [117]. Using a rat model, Nehra et al. quantitated POH (and perillic acid) levels in plasma and cerebrospinal fluid (CSF) after intranasal and intravascular POH delivery [118]. Very interestingly, this group was able to demonstrate that intranasal administration of POH resulted in tenfold higher CSF-to-plasma ratios for POH and perillic acid as compared to what was achieved after an equal dose of POH was administered intravascularly. While neuro-PK data are not yet available from patients, the results from these preclinical studies further indicate that intranasal administration of POH results in direct nose-to-brain delivery, further underscoring the potential value of this mode of drug delivery for brain cancer therapy.

### 4.2. Intranasal POH as a Co-Delivery System

Co-delivery systems for drug delivery come in many designs. They may comprise carriers, where one or more drugs are packaged into liposomes, nanoparticles, hydrogels, dendrimers, or other vehicles [119,120], and some of these are being developed specifically for the purpose of intranasal delivery to reach the brain via the nose-to-brain route [121,122]. Studies with POH have also revealed characteristics that make this monoterpenoid suitable for the purpose of transporting other drugs directly to the brain via the nose-to-brain route.

For instance, the intranasal delivery of NEO100, in combination with bortezomib, was investigated in rodent models of GBM [123]. Bortezomib (Velcade^®^) is a proteasome inhibitor that was approved to treat multiple myeloma and mantle cell lymphoma [124]. Its penetration of the BBB is extremely poor [125], which is consistent with its demonstrated lack of therapeutic effect in mouse GBM models [126,127] or when tested in the clinic with recurrent glioma patients [128,129,130] after intravenous delivery. In stark contrast, bortezomib showed high activity in mouse models of orthotopic GBM when it was delivered directly into the tumor using an Alzet pump [127], and at low nanomolar concentrations against glioma cells in vitro [131,132,133]. Collectively, these observations indicate that, in principle, bortezomib can be highly effective against GBM, but in reality, this benefit cannot be realized due to the agent’s inability to penetrate the BBB. In an attempt to circumvent this obstacle, bortezomib was mixed with NEO100 and delivered intranasally to mice harboring xenografted human GBM cells in their brains. Results from this study [123] demonstrated that the co-delivery of bortezomib with NEO100 significantly prolonged the survival of tumor-bearing animals as compared to untreated mice, mice receiving intranasal bortezomib in the absence of NEO100, or mice receiving bortezomib via intravenous infusion. Furthermore, the authors were able to demonstrate substantially greater amounts of bortezomib in the cerebrospinal fluid (CSF) of rats after treatment with the bortezomib/NEO100 combination than after treatment with bortezomib alone. Together, these studies provided proof of principle that a pharmacologic agent with very poor BBB penetration can effectively be delivered to the brain by using a NEO100-based formulation and intranasal delivery, where the amphiphilic physicochemical properties of NEO100 serve to increase permeation of both components through the BBB and cell membranes.

## 5. Intra-Arterial Perillyl Alcohol

While intranasal applications of POH and NEO100 exploit direct nose-to-brain access routes and thereby circumvent the obstacle placed by the BBB, an intra-arterial path of administering NEO100 has provided evidence that it also can directly confront the BBB and “open” it, i.e., diminish its barrier function to enable mostly unrestricted permeation of otherwise BBB-impermeable compounds. This procedure is being explored and characterized in preclinical models and might have certain advantages over the currently established clinical method of opening the BBB with mannitol.

Intracarotid injection of mannitol has been used for the past three decades as a means to reversibly open the BBB. Its disruptive mechanism is based on the contraction of endothelial cells and the resulting widening of tight junctions as a result of the hyperosmotic impact of high concentrations of this sugar alcohol. However, this procedure has led to variable responses in BBB breakdown, seizures, risk of brain embolism, and catastrophic bleeds [134,135]. As a result, the use of this procedure is restricted to specialized centers, has not found widespread acceptance, and does not lend itself to use in general clinical practice. Similarly, the effects of radiation on BBB permeability have long been recognized [136] and are thought to impact BBB integrity through the killing of endothelial cells and resulting neuro-inflammatory reactions [137]. However, many details, including the time course and magnitude of increased opening, remain to be established [138]. A more recently introduced BBB disruption technique is transcranial-focused ultrasound coupled with intravenously delivered microbubbles [139], which has advanced to clinical trials [140,141]. This procedure disrupts the BBB through cavitation and acoustic forces, leading to reduced expression of tight junction proteins, along with an inflammatory response. However, it represents a rather invasive procedure because the skull needs to be opened in order to introduce the ultrasound probe that triggers BBB permeation. Overall, despite progress in the development of new procedures, the need for safer, more efficient, and reversible BBB-opening methods persists [142].

Based on the use of preclinical models, intra-arterial injection of NEO100 was very recently presented as a novel BBB-disruptive method [143,144]. In these studies, intra-arterial injections of NEO100 were accomplished via ultrasound-guided intracardiac injections in mouse models, which resulted in rapid BBB opening that lasted up to four hours. During this window of opportunity, intravenously delivered agents were able to effectively enter the brain. For instance, Evans blue present in the systemic circulation was shown to prominently stain mouse brains blue after NEO100-induced BBB opening. This effect was not achieved via intra-arterial injection of a vehicle without NEO100, nor in cases where NEO100 was injected intravenously instead of intra-arterially (possibly due to dilution by the circulatory system and surrounding tissue). The mechanism of action of NEO100-induced BBB opening was demonstrated to be secondary to transient downregulation of specific gap junction proteins, resulting in short-term, fully reversible breakdown of barrier function [143,144]. Similar outcomes were seen with the non-BBB-permeable anticancer drug methotrexate, which accumulated to high levels in brain tissue and CSF after NEO100-induced BBB opening. Intriguingly, increased brain entry was not limited to small therapeutic molecules, such as methotrexate, but could also be documented for therapeutic antibodies, e.g., a checkpoint-inhibitory anti-PD1 antibody and the anti-HER2 antibody trastuzumab, and even for whole cells, using chimeric antigen receptor (CAR) T cells as a model [144]. Furthermore, these authors provided proof of principle that NEO100-based BBB opening is able to achieve therapeutic benefit in mouse models of intracranially implanted HER2+ breast cancer cells [143]. Mice harboring such tumors were subjected to a single round of intra-arterial injection of NEO100, along with intravenous delivery of trastuzumab or the trastuzumab-drug conjugate ado-trastuzumab emtansine (T-DM1; Kadcyla^®^). There was a tumor-selective accumulation of these therapeutic antibodies in the brain and significantly prolonged survival of mice receiving this treatment.

With regard to translating the above preclinical results to patient applications, it was proposed [143] that patients with intracranial malignancies would be subjected to standard interventional neuroradiology that would allow for selective delivery of NEO100 to tumor-feeding intracranial arteries. Intra-arterial injections would be performed via cannulation of the femoral artery with selective threading toward one of the cerebral arteries. This transfemoral approach [145,146,147] represents a common, straightforward procedure in a variety of clinical settings and is generally considered a safe procedure [148,149,150]. It is routinely performed by endovascular neurosurgeons in the context of cerebral angiograms, aneurysm coiling, tumor embolization, and thrombectomies [151]. Combining this transfemoral approach with NEO100 as the injectate has not yet been tested in the clinic. However, if the safety of transient BBB opening with this approach could be confirmed, it would have far-reaching implications. It would provide a significantly safer alternative to intracarotid mannitol, its use would not be restricted to specialized medical centers, and its application would expand to include the treatment of many neurological diseases beyond intracranial malignancies.

## 6. Perillyl Alcohol Derivatives, Analogs, and Conjugates

Based on the recognition of POH’s suitable physicochemical properties in combination with its anticancer activity, numerous studies have explored whether alterations and modifications of this molecule might result in enhanced therapeutic activity. For instance, the glycosylation of drugs can often significantly alter their pharmacological properties and ADMET (absorption, distribution, metabolism, excretion, and toxicity) [152]. Glycosides of POH exist in nature and some of them were initially characterized as aldose reductase inhibitors [153]. Additional unnatural POH derivatives were synthesized and tested for their potential anticancer activity [154,155,156]. For instance, Nandurkar et al. [155] generated a group of 34 POH glycosides that were tested for their antiproliferative activity against two established cancer cell lines in vitro. This group identified several new compounds that exerted greater in vitro cytotoxicity than POH, with POH 4′-azido-D-glucoside as the most potent POH adduct. Similarly, a series of cyclodiprenyl phenols were synthesized from perillyl alcohol and synthetic phenols, and some of these meroterpenes showed strong antiproliferative and apoptosis-inducing activity against three different cancer cell lines in vitro [157]. POH was also complexed with β-cyclodextrin, which resulted in improved anticancer potency in vitro and in a sarcoma S180-induced mouse model in vivo [158]. Beyond these selected examples, several more reports on modified POH (or perillic acid) molecules exist, and the interested reader is referred to excellent recent reviews [159,160] for additional information and detailed references.

Some hybrid drugs are generated as multifunctional compounds, where two or more molecules with already well-characterized pharmacologic activity are conjugated through a stable or metabolizable linker. The central rationale of associating these pharmacophores is to increase their potency, to possibly enable overall dosing at lower levels to minimize toxic side effects, and to reduce the likelihood of emerging drug resistance [161,162]. For example, it was investigated [163] whether linking POH to dihydropyrimidinones would generate increased anticancer impact. Dihydropyrimidinones are important heterocyclic compounds and, like POH, have revealed a large scope of pharmacological activities, including anticancer properties [164]. Fifteen novel perillyl–dihydropyrimidinone compounds were generated and some of them were found to effectively kill established ovarian cancer, melanoma, and glioblastoma cell lines, but spared a normal keratinocyte cell line [163]. The cytotoxic IC_50_ ratio between the keratinocyte cell line and the glioblastoma cell line was about 50, meaning that 50-fold higher drug concentrations were required to kill the normal cells as compared to the tumor cells, indicating promising cancer cell-selectivity of some of these novel compounds.

### 6.1. Conjugation with POH to Enhance Permeability

In a different approach toward generating POH-based multifunctional compounds, the group of Thomas Chen at the University of Southern California sought to exploit the amphipathic nature of POH to modify existing drugs toward increased penetration of biological barriers, in particular the BBB. Using software from Advanced Chemistry Development, Inc. (ACD Labs, Toronto, ON, Canada), to predict BBB penetration and brain entry of a given molecule, they performed in silico characterization of novel molecules, where POH was conjugated to pharmacologic agents of interest with the objective to identify therapeutics with superior ability to enter the brain.

#### 6.1.1. POH Conjugated to Temozolomide (NEO212)

**Figure 4:**
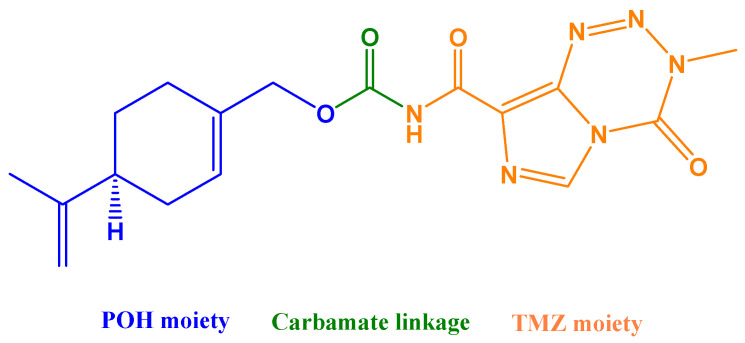
Chemical structure of a TMZ–POH conjugate (NEO212). NEO212 was generated by covalent conjugation of POH (perillyl alcohol; **blue**) to TMZ (temozolomide; **orange**) via a carbamate linkage (**green**).

The so-far best-characterized hybrid molecule emerging from in silico studies of BBB penetration is NEO212 (Figure 4), consisting of POH covalently conjugated via a carbamate bond to TMZ, the alkylating agent in clinical use for GBM. NEO212 has been investigated in over a dozen studies that used a variety of preclinical in vitro and in vivo tumor models and consistently established the robust anticancer activity of this novel molecule, along with low toxicity [165,166,167,168] and superior ability to penetrate the BBB [169].

Among the remarkable results was the finding that the NEO212 hybrid molecule exerted greater anticancer activity than an equimolar mix of its individual constituents, i.e., combining POH with TMZ—akin to the conventional combination therapy mode—was unable to achieve the same tumoricidal potency in vitro or in vivo as the conjugated molecule, indicating that the activity of the conjugated compound was greater than the sum of its parts [165,166,167,168,170]. For example, the effects of NEO212 were investigated in mouse brain tumor models of GBM [170,171] or intracranial breast cancer (to mimic brain-metastatic conditions) [165]. It was demonstrated that oral treatment with NEO212 exerted greater therapeutic activity than equivalent doses of TMZ or POH, either individually or when combined. When compared side by side and given at equal doses, the amount of NEO212 measured in brain tissue was threefold higher than the amount of TMZ [169], confirming the in silico results, where NEO212 was predicted to cross the BBB more efficiently than TMZ.

NEO212’s superior penetration ability was not restricted to having the BBB as the target. Cellular membranes present important biological barriers too, and here, NEO212 revealed beneficial characteristics as well. In a key in vitro experiment, it was measured how much TMZ was present inside cells after treatment of cells with equal concentrations of either NEO212 or TMZ [169]. The results showed >10-fold higher intracellular TMZ levels when cells were treated with NEO212 as compared to treatment of cells with TMZ [169]. Similarly, the expected metabolite of TMZ breakdown, the relatively stable 5-aminoimidazole-4-carboxamide (AIC), was correspondingly elevated as well, indicating that NEO212-treated cells generated substantially more of the active, DNA-alkylating species than TMZ-treated cells. Overall, these observations are consistent with a model where the conjugation of POH to TMZ enables superior barrier penetration and consequential enhancement of the anticancer impact of the TMZ partner molecule, qualifying POH as a potent permeation enhancer.

Based on the highly efficient cellular uptake of NEO212, it was hypothesized [167] that NEO212 might also exert a beneficial impact on leukemic cancer types, where the encounter between drug and tumor cells might be more immediate and most conducive to optimal drug uptake. The veracity of this conjecture was tested in acute myeloid leukemia (AML) cells in vitro and in vivo, which included AML cell lines that were strongly resistant to cytosine arabinoside (AraC), the most commonly used chemotherapeutic agent for this disease [172]. The in vitro experiments showed potent cytotoxic activity of NEO212 at low micromolar concentrations, along with confirmation that TMZ and its breakdown product AIC were present at high levels inside NEO212-treated AML cells. The most striking results were generated when AML cells were implanted into immunocompromised mice, followed by short cycles of NEO212 treatment. In these in vivo experiments, the majority of NEO212-treated animals survived until the end of the observation period (300 days), while all vehicle-treated control animals succumbed to disease within a few weeks [167]. The same outcome was achieved with AraC-resistant AML cell lines. Although the authors did not verify whether the NEO212 treatment was eradicative, i.e., whether residual, potentially dormant tumor cells were still present after 300 days, they concluded that treatment was curative, because the long-term survivors continued to thrive in the absence of any signs of disease.

The strikingly impressive therapeutic activity of NEO212 in this preclinical leukemia model is consistent with the above-stated assessment of POH as a permeation enhancer, in this case, for conjugated TMZ as the partner. However, it raises the question of tumor specificity and toxic side effects because normal cells—bone marrow cells and white blood cells (WBC) in particular—might be as susceptible as tumor cells. In the case of TMZ, for example, myelosuppression is a well-recognized, dose-limiting side effect of treatment [173]. Surprisingly, however, detailed analysis of complete blood counts (CBC) from NEO212-treated mice, together with histopathology of bone marrow and several other organs, failed to reveal signs of toxicity in these compartments [165,166,167,168]. Instead, NEO212 appeared to be very well tolerated, even during extended treatment periods and escalated dosages [167]. While this wide therapeutic window observed in these preclinical studies bodes well for future clinical applications, the underlying determinants of tumor selectivity of NEO212 remain to be established.

#### 6.1.2. POH Conjugated to Rolipram or 3-Bromopyruvate or Valproic Acid

Beyond NEO212, other POH-based conjugates emerged from in silico analysis using ACD Lab’s software that predicted greater BBB penetration and possibly increased membrane permeation. NEO214 is a conjugate of POH and rolipram, a selective phosphodiesterase 4 (PDE4) inhibitor that was in clinical development as an anti-depressant in the 1990s. Although rolipram was never approved for marketing [174], it was considered for repurposing as an anticancer agent based on its recognized beneficial activity in preclinical cancer models [175,176]. In mouse models of intracranial glioblastoma, NEO214 revealed significant therapeutic activity [177], indicating that it was indeed able to effectively cross the BBB, as was predicted from the in silico analysis. Intriguingly, as was observed with NEO212, combination therapy with the individual components, i.e., POH mixed with rolipram, was unable to mimic the much stronger anticancer effects of the conjugated NEO214 molecule [177,178].

Another interesting observation was made with NEO218, a conjugate of POH and 3-bromopyruvate (3BP), a synthetic halogenated derivative of pyruvate that was also investigated as an anticancer agent [179]. Cellular uptake of 3BP is strictly dependent on the presence of a transmembrane transport protein mono-carboxylate transporter 1 (MCT-1), where cells lacking MCT-1 are resistant to the toxic impact of 3BP [180]. It was demonstrated that applying selective pressure to MCT-1-positive HCT116 colon carcinoma cells by exposing them to 3BP in vitro resulted in the elimination of transporter-positive cells, with the simultaneous emergence of an MCT-1-negative sub-population that displayed resistance to 3BP [181]. In comparison, NEO218 exerts its cytotoxic impact in the absence of MCT-1, i.e., the conjugation of POH to 3BP enabled cellular 3BP import across the cytoplasmic membrane without the need for an active transport mechanism. Consequently, treatment of HCT116 cells with NEO218 did not result in emergent resistant clones, because the loss of MCT-1 expression could not provide a survival advantage [181], providing an example of POH’s applicability to minimize the emergence of cancer cell drug resistance during therapy.

Yet another conjugate, NEO216, which is POH covalently linked to valproic acid (VPA), was preliminarily characterized. Compared to VPA, a well-known inhibitor of histone deacetylase (HDAC) activity [182], NEO216 showed reduced HDAC inhibition properties but revealed an ability to inhibit 4-aminobutyrate aminotransferase (ABAT), a key enzyme responsible for the catabolism of the neurotransmitter gamma-aminobutyric acid (GABA) [183]. This novel function has made NEO216 useful for a recent study that established the role of GABA metabolism in leptomeningeal dissemination of medulloblastoma [183] and more applications of this novel conjugate are anticipated.

### 6.2. POH and Penetration of the Skin

Similar to intranasal delivery, transdermal drug delivery (TDD) is being explored as an alternative route to other modes of drug administration, as it too has certain advantages that include the avoidance of hepatic first-pass drug metabolism, reduced GI side effects, and good patient compliance. The key barrier to efficient TDD is presented by the stratum corneum, the outermost layer of the epidermis that can severely limit percutaneous drug absorption [184,185]. To overcome this barrier, a great variety of chemical compounds have been investigated for their use as potential skin penetration enhancers, i.e., as promoters or accelerants to enable and increase the transdermal flux of topically applied pharmacologic agents (see details and further references in excellent reviews [186,187]). While many terpenes were identified as potential skin penetration enhancers [188,189], POH stands out among them because it also appears to exert beneficial transdermal activity on its own, i.e., without the need for other co-transported therapeutic agents.

For instance, when applied topically to the skin, POH was able to inhibit tumor development in the skin of mice exposed to the carcinogen DMBA [190,191] or to ultraviolet B radiation [192]. These preclinical observations prompted clinical studies to investigate whether topical application of POH might prove effective as a skin cancer chemoprevention strategy. A Phase I study enrolled participants with normal, healthy skin who applied POH, formulated as a cream [193], to their forearms twice daily for 30 days. The results showed that the POH cream was well tolerated and no cutaneous or systemic toxicities were observed [194]. In a subsequent Phase IIa study with POH cream and twice-daily applications to the forearms of participants over three months, it was investigated whether this treatment could reverse actinic (sun) damage. Modest effects of POH in sun-damaged skin could be documented, but the overall outcome was unimpressive [195], and no follow-up studies have been reported since. In other investigations, the authors used two murine models of dermatitis (induced by 12-O-tetradecanoylphorbol-13-acetate (TPA)) and of mechanical skin lesions (induced by epidermal incisions) that received daily treatments with topical POH prepared in sunflower oil. These studies showed that POH exerted significant anti-inflammatory effects along with improved tissue repair and wound healing [196]. Related observations were made with a mouse model of neuronal injury (although, in these cases, POH was given orally), where POH reduced gliosis and improved sensory and motor function recovery [197].

Among the above-introduced POH hybrid molecules, NEO412 was designed as a topical treatment for melanoma in situ (MIS). Although MIS has a better prognosis than metastatic melanoma, its effective treatment is complicated by ill-defined margins during surgical removal of the lesions, potentially leading to invasive melanoma [198]. Therefore, a topical treatment that would target unrecognized malignant components that might remain after surgery could be of great benefit to minimize the risk of recurrence. NEO412 is a tripartite agent consisting of three bioactive agents: POH, TMZ, and linoleic acid, with the latter representing a well-established transdermal penetration enhancer [199,200]. In vitro studies confirmed that NEO412 was able to effectively kill melanoma cells through the alkylation effect of TMZ [201]. In a mouse model of subcutaneous melanoma, topical application of NEO412 increased tumor cell death, along with decreased neo-angiogenesis in tumor tissue. Intriguingly, these effects could also be observed in tumors that were located distant from the skin treatment site, indicating that topical NEO412 was able to enter the circulation and reach tumor cells that might have metastasized already [201]. Although it remains to be established how much each penetration enhancer, i.e., POH vs. linoleic acid, contributed to transdermal migration of the tripartite molecule, the significant anti-melanoma effect that was achieved clearly indicates the benefit of this combination.

## 7. Conclusions and Outlook

POH is a monoterpenoid with an amphipathic character that appears to provide the physicochemical basis for its multifaceted applicability to overcome biological barriers. By means of further purification and CGMP manufacture, an effective pharmaceutical agent, namely, NEO100, was realized from POH that demonstrates good therapeutic prospects from in vitro, in vivo, and clinical testing. It can circumvent the BBB when administered via the intranasal route or physically open the BBB when given intra-arterially. It easily penetrates the cytoplasmic membrane without the need for cell surface transport proteins to facilitate its cell entry. In addition, it appears to harbor skin-penetrating potency. While much research has focused on characterizing the anticancer activity of the POH molecule, other avenues of investigation have further established its ability to enhance the therapeutic impact of different pharmacologic agents, either through enabling the transport of BBB-impermeable drugs via the intranasal route directly into the brain or through its covalent conjugation to specific drugs that results in overall increased penetration of the above-described biological barriers by the conjugated molecule.

The general principles attributed to the use of POH for increasing permeability across various biological barriers represent platform technologies that could be applicable to multiple clinical needs and situations. Although the bulk of applications is currently outlined for the treatment of primary and metastatic brain tumors, these POH-based concepts are adaptable for use in therapies aimed at neurodegenerative and infectious conditions of the brain. Moreover, the variety of molecules that could be delivered to the brain with the use of these platforms is not restricted to established drugs, but may include nucleic acids, i.e., siRNA, miRNA, and mRNA, as well as therapeutic proteins and antibodies, and possibly even intact cells and stem cells.

## Data Availability

Not applicable.

## Abbreviations

API	Active pharmacologic ingredient
BBB	Blood–brain barrier
CGMP	Current Good Manufacturing Practices
CNS	Central nervous system
GBM	Glioblastoma
IDH	Isocitrate dehydrogenase
MRI	Magnetic resonance imaging
POH	Perillyl alcohol
RT	Radiation therapy
TMZ	Temozolomide
